# The enigmatic historical record of the leaf beetle *Acentroptera norrisii* in Spain: failed biological introduction or curatorial artefact?

**DOI:** 10.7717/peerj.21213

**Published:** 2026-06-18

**Authors:** Adrián Sánchez Albert, José Antonio López-Sáez, Inés Modolell, Peter Lawrence, Mercedes París

**Affiliations:** 1 Entomology Collection, Museo Nacional de Ciencias Naturales (MNCN-CSIC), Madrid, Comunidad de Madrid, Spain; 2 Environmental Archaeology Research Group, Institute of History (CCHS-CSIC), Madrid, Comunidad de Madrid, Spain; 3 Cambridge University Hospitals (CUH) Foundation Trust, Cambridge University, Cambridge, United Kingdom; 4 Department of Zoology, Cambridge University, Cambridge, United Kingdom

**Keywords:** *Acentroptera norrisii*, Cassidinae, Chrysomelidae, Coleoptera, Biological introduction, Mislabelling, Museum specimen, Historical palynology, SEM analysis, Spain

## Abstract

**Background:**

Natural History Collections (NHC) often contain exceptional specimens documenting unusual historical events, and these records can be valuable for detecting overlooked biological introductions. Here, we examine a striking case: a Neotropical leaf beetle, *Acentroptera norrisii*, labelled as collected in central Spain in 1998 by Dr J. Modolell. Given the species’ native distribution in the Neotropics, this record raises the question of whether it represents a failed historical introduction or a curatorial artefact.

**Methods:**

We reconstructed the circumstances of the collection event using the collector’s personal archives. We conducted scanning electron microscopy (SEM) to document microscopic particles on the specimen’s cuticle. We also analysed the content of the original specimen box to evaluate the likelihood of pollen cross-contamination, considering the geographical origin and family-level identity of all co-stored specimens.

**Results:**

Archival evidence confirms the collector’s presence at the labelled locality and date. SEM revealed several pollen grains consistent with plant taxa occurring at the collection site. However, the original box also contained numerous Spanish specimens belonging to beetle families with known anthophilous habits, meaning that cross-contamination during storage cannot be fully excluded. As a result, the palynological evidence remains inconclusive.

**Conclusions:**

Together, the available evidence makes a failed historical introduction a plausible scenario, yet the inconclusive particle analysis prevents any confident confirmation. This case highlights both the potential and the limitations of NHC-derived data in invasion biology: while museum collections can preserve traces of otherwise “invisible” introductions, interpreting isolated and context-poor specimens remains inherently uncertain.

## Introduction

Natural History Collections (NHCs) are widely recognised as primary sources of biodiversity data (Suarez & Tsutsui, 2004; Pyke & Ehrlich, 2010; Holmes et al., 2016; Meineke et al., 2018; Kharouba et al., 2019; Popov et al., 2021; Alofs et al., 2025), providing essential baselines for reconstructing past communities, interpreting ongoing ecological trends, and even glimpse possible future scenarios for life on Earth (Guralnick & Van Cleve, 2005; Navarro et al., 2025).

When looking back in natural history through preserved specimens (*i.e.,* historical ecology, Szabó, 2014; Wood & Vanhove, 2022; Navarro et al., 2025), ecologists can face some limitations, inherent to the opportunistic and biased nature of specimen collection (Guralnick & Van Cleve, 2005; Pyke & Ehrlich, 2010; Kharouba et al., 2019; Meineke & Daru, 2021). In terms of the specimen’s spatiotemporal context, associated data can be absent, incomplete or even offer erroneous evidence (*i.e.,* type I/α error or false positives)—especially when there is a main source of capture conditions (*e.g.,* labels)–, and undoubtedly, the scientific value of specimens ultimately depends on the accuracy of these data (Boessenkool et al., 2010; Wehi, Whaanga & Trewick, 2012; Fearing et al., 2025).

Discerning between errors and outliers (*i.e.,* specimens collected out of its known geographical range) in specimen data can be extremely challenging. Researchers and other museum staff typically use alternative sources of information (*e.g.,* collectors’ field notes, photographs, oral testimonies, *etc*.) and in-depth inspection techniques (*e.g.,* microscopy examination, Computed tomography (CT)-scanning, DNA and other chemical analysis, *etc*.), to discard or confirm records (Askevold, 1991; Rasmussen & Prŷs-Jones, 2003; Boessenkool et al., 2010; Wehi, Whaanga & Trewick, 2012; Rossini, Vaz-de Mello & Zunino, 2016). Given the crucial role of NHCs data in understanding past and present of biological invasions (Holmes et al., 2016; Kim et al., 2023), re-examining such records is crucial.

Given this premise, in this work we aimed to critically assess the evidence surrounding the Spanish record of a tortoise leaf beetle (*Acentroptera norrisii* Guérin-Méneville, 1844; see Staines, 2014) collected by Dr Juan Modolell and currently housed at the Entomology Collection of the Museo Nacional de Ciencias Naturales (MNCN-CSIC). The hypothetical past presence of this specimen in the Iberian Central System (Guadarrama mountain range)—well outside the known neotropical range of the genus—, raises the question of whether this record represents a case of mislabelling or a failed introduction event.

To address this case, we dive into the circumstances surrounding the specimen’s collection event, and explore the possibility of its introduction through the evidence of microscopic particles adhering to its cuticle. Our analysis illustrates both the value and the limitations of historical specimens in reconstructing species invasions, and underscores the need for critical interpretation of exceptional historical records from museum collections.

## Materials & Methods

### Discovery of the specimen

Dr Juan Modolell Mainou (Barcelona, 1937) was a renowned Spanish molecular biologist and insect enthusiast who sadly passed away in 2023. Parallel to his professional career in protein biosynthesis and developmental biology (Ruiz-Gómez & Campuzano, 2023), he cultivated a personal entomological collection consisted in more than 5,000 specimens (mostly butterflies, ∼84.17%) from more than 50 countries around the world. Its collection was donated to the MNCN-CSIC Entomology Collection dependencies in September 2023.

**Figure 1:**
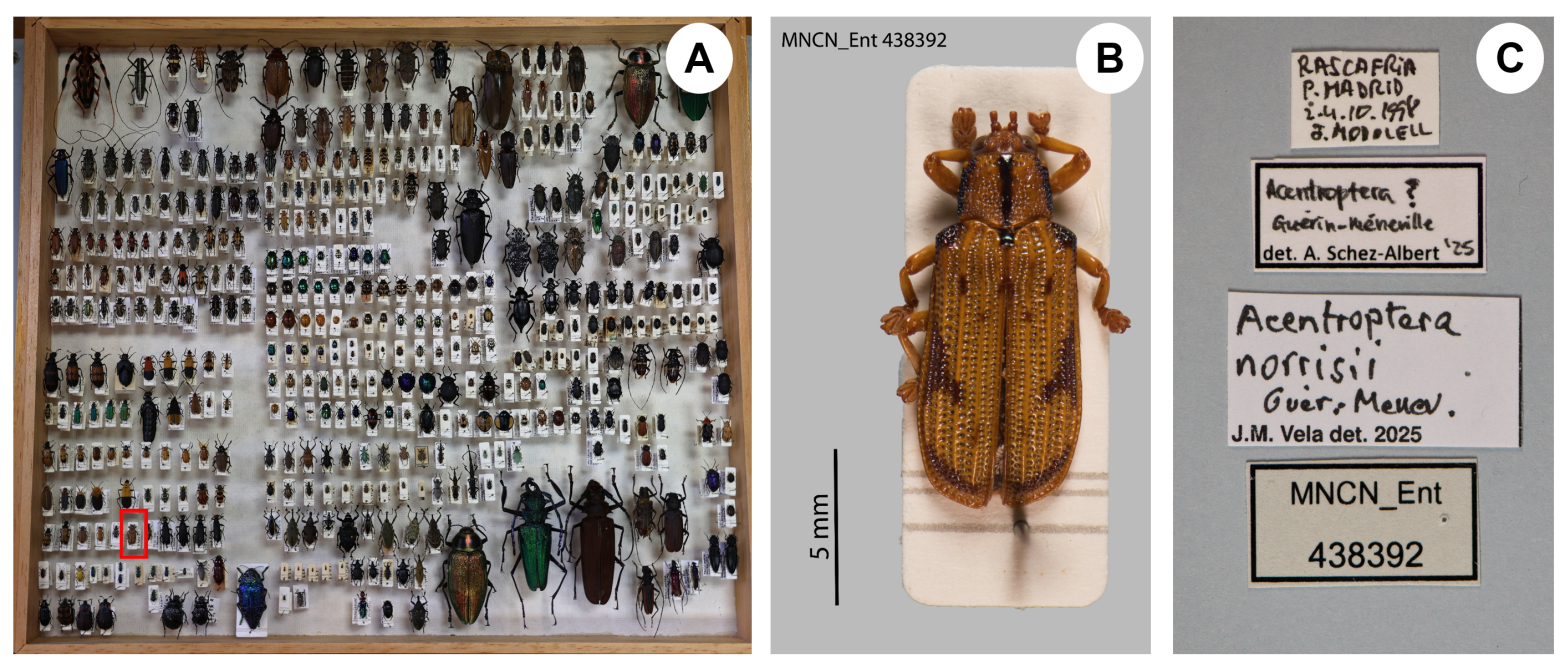
*Acentroptera norrisii* specimen (MNCN_Ent 438392) housed at MNCN-CSIC (A–C). (A) Whole-drawer photography of the original box containing the specimen under scrutiny, framed in red. Photo credit: Lara García Palencia. (B) High-resolution dorsal image view of the specimen’s habitus. Photo credit: Adrián Sánchez Albert. (C) Specimen’s associated labels. Photo credit: Adrián Sánchez Albert.

When assisting in processing the coleopteran specimens of Dr Modolell’s collection in June 2025 a reputed coleopterologist and consultant of the collection (Iñaki Recalde), singled out several specimens, among several dozens of beetles in the original box (no. 32), as particularly intriguing due to its unfamiliar appearance. After a closer inspection under binocular lens, he drew the first author’s attention on the capture conditions of one particular specimen (MNCN_Ent 438392). This specimen was pinned and glued to a label and, according to its data label, it was collected in Rascafría (North of Madrid province, Central Spain), between 2–4 October 1998 by Dr Modolell himself (Fig. 1).

### Taxonomic identification

The specimen lacked its antennae and right posterior leg, and its anterior right leg is broken, lacking two tarsomeres. However, the specimen could be reliably identified by Dr J. M. Vela López as *Acentroptera norrisii* Guérin-Méneville, 1844 during his visit to the MNCN-CSIC Entomology Collection dependencies in July 2025. Very little is known about the biology of this species, but its known geographical distribution is restricted to Brazil and French Guiana (Staines, 2014). Therefore, the capture location specified by Dr Modolell appeared unusual.

### Archival and historical investigation

To verify the authenticity of the specimen label and the plausibility of the record, we conducted a thorough contextual investigation. Dr Modolell’s descendants and a former colleague were contacted to clarify his collecting activities during the late 1990s through further tracks that could confirm his visit to the capture locality in that date (such as images, field notes or travel tickets). Personal archives were examined for travel records or locality references matching the label data.

In addition, the locality indicated on the specimen label (Rascafría, Madrid Province) was visited to confirm potential sources of introduction. The former owner of the locality’s greenhouse was interviewed to determine whether plant material of Neotropical origin might have been imported around the time of collection.

### Scanning Electron Microscopy examination

The specimen was examined under MNCN-CSIC scanning electron microscope (SEM, model FEI Inspect S50; FEI Company, Hillsboro, Oregon, USA) to detect possible adhered particles such as pollen or debris. Prior to imaging, the specimen was handled minimally and not cleaned nor coated to preserve any surface residues. Micrographs were taken under vacuum (0.46 Torr) from a dorsal viewpoint, covering several body regions in anteroposterior sequence (head, thorax and elytra). Multiple images at different magnifications were obtained to document the general distribution and detail morphology of surface particles.

### Associated specimens and assessment of cross-contamination risk

To evaluate the potential for pollen cross-contamination from other specimens originally sharing the same box, these were identified to at least the family level to detect taxa whose adults can be potential pollen donors. In addition, their geographical origins were recorded to establish the relative proportions of Spanish and foreign material that could have contributed to any pollen transfer.

## Results

**Figure 2:**
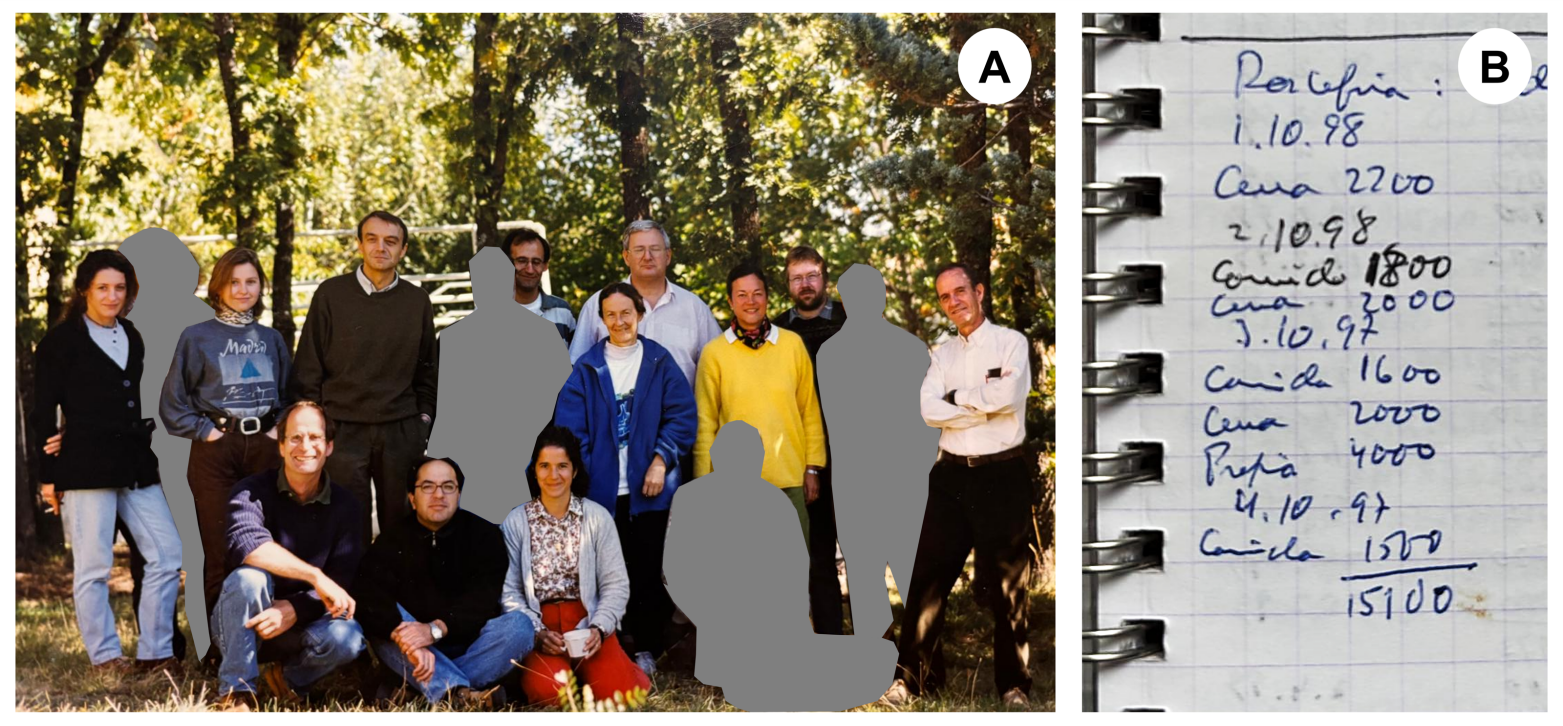
Documentation of Dr J. Modolell’s presence at the specimens’ collection site (A–B). (A) Group photo taken at the October 1998 meeting in Rascafría. Dr Modolell is standing on the far right wearing a white shirt. Grey shapes correspond to people for whom we could not obtain image consent. Courtesy of Peter A. Lawrence. (B) Scan of the logbook’s footnote attesting expenditures of Dr Modolell’s travel to the locality, expressing several lunch expenses from 2–4 October 1998. Image credit: Inés Modolell Mainou.

Within Dr Modolell’s personal archives, his 1998 field logbook recorded expenses associated with several trips: 28 May–18 August to Cambridge (United Kingdom), 21–27 August to Tenerife (Canary Islands), 12–18 December to Chile, and additional travels within the Iberian Peninsula. Among these was a laboratory meeting held in Rascafría (1–4 of October 1998; Fig. 2A), the locality indicated on the specimen’s label. This trip was further corroborated by Peter A. Lawrence’s photographic archive (Fig. 2B). The confirmation of Dr Modolell’s presence at Rascafría in that date supports the authenticity of the capture conditions indicated on the label.

Furthermore, the interview with the former proprietor of the local greenhouse facility supported the presence of translocation-competent botanical specimens (*e.g.,* bromeliads) within the specific spatio-temporal context in which the introduction event could have occurred.

The microscopic analysis took approximately two hours of SEM inspection of the specimen’s dorsal surface, during which fifteen microscopic particles were recorded. Thirteen of these were recognised as pollen grains, while the remaining two were identified as a diatom (probably *Planothidium*, Achnanthidiaceae) and a lepidopteran wing scale of uncertain identity. Of the thirteen pollen grains, four morphotypes could not be identified: two because they were collapsed and other two because of lack of diagnostic traits. The remaining seven were tentatively assigned to *Pinus sylvestris* L. type (*N* = 1), Poaceae Barnhart (*N* = 4) and Liliaceae Juss. (*N* = 2). The specimen’s cuticular ornaments were covered in what appeared to be plant remains and soil debris, with pollen deeply embedded within them.

The content analysis of the original box yielded a total of 469 beetle specimens (and three hemipterans) belonging to at least 20 taxonomic families (Fig. 2A; Table S1). Of these, 194 specimens (41.4%)—representing 18 families—were collected in Spain (Iberian Peninsula and insular territories), while the remainder originated from other regions or lacked provenance data.

## Discussion

The unexpected historical presence of a Neotropical tortoise leaf beetle in Spanish territory raises a central question: does this *Acentroptera norrisii* specimen represent a failed introduction or a curatorial artefact? We discuss the plausibility of a past failed introduction and the limitations of the available evidence by reviewing the existing proofs and placing this record within the broader context of insect invasions.

### Six-legged invasions: the case of leaf beetles

Insects, after plants, constitute the second-largest group of non-native organisms worldwide (Roques, 2010; Seebens et al., 2017; Mally et al., 2025). Beetles (order Coleoptera), the most species-rich insect order, greatly contribute to the global pool of non-native insect insects (Roques, 2010; Mally et al., 2025). Beyond taxonomic identity, herbivory appears to be an ecological trait favouring insect translocation outside native ranges (Mally et al., 2025).

Europe, and mainland Spain in particular, is a major hotspot for introductions of alien terrestrial arthropods (Roques, 2010; Turbelin, Malamud & Francis, 2017; Soto et al., 2025). The end of the 20th century saw a marked acceleration of new records (Roques, 2010; Soto et al., 2025), although this rate has begun to slow for Coleoptera in recent decades (Mally et al., 2025).

Within Coleoptera, leaf beetles (Chrysomelidae)—as *A. norrisii*—rank among the most frequently translocated families (Beenen, 2005; Beenen & Roques, 2010; Liebhold et al., 2021; Mally et al., 2025). At least 27 historical translocations of tortoise leaf beetles (Chrysomelidae, Cassidinae) have been documented (see Beenen, 2005).

### Out of the green: a probable introduction pathway

Species of Acentroptera are mainly associated to Bromeliaceae, laying their eggs on the leaves and developing within these plants (see Staines, 2014; Albertoni & Casari, 2017). The widespread use of bromeliads as ornamentals (Negrelle, Mitchell & Anacleto, 2012) makes these beetles plausible candidates for unintentional translocation *via* international plant trade (Beenen, 2005; Roques, 2010; Gippet et al., 2019).

Despite the exact host plant of *A. norrisii* remains undescribed and unknown (but see Staines, 2014), the escape from human facilities and transport-related mechanisms (*i.e.,* contaminant and stowaway pathways) would be the most probable (Rabitsch, 2010; Turner et al., 2021; Fenn-Moltu et al., 2023; Soto et al., 2025). Testimonial information confirms that a plant nursery was active at the locality, commercialising bromeliads, at the time of collection (J Cachofeiro, pers. comm., 2025), thus suggesting the possibility that the beetle escaped from these greenhouse facilities (Wang et al., 2015), and then collected by Dr J. Modolell.

### The beetle in the room: overlooked insect invasions

As Orlova-Bienkowskaja (2016: 318) observed, “in the level of knowledge of invasions, entomology drops behind many other fields of biology”. This idea is reinforced by recent global assessments, which highlight major data gaps and persistent uncertainty in the documentation of alien insect distributions (Seebens et al., 2025). This fundamental limitation raises the problem of ‘known unknowns’: how many insect introductions are we simply missing? (*cf.* Jeschke & Pyšek, 2018; Turner et al., 2025). Indeed, the arrival and even establishment of alien beetle species can pass unnoticed, as previously documented for bark beetles in Europe (Kirkendall & Faccoli, 2010). Unintentional introductions may remain invisible until the species becomes established, whereas intentional but unsuccessful introductions tend to be recorded (Beenen, 2005). Thus, overlooked insect introductions are entirely plausible (‘invasion tens rule’, Jeschke & Pyšek, 2018).

This is precisely where Natural History Collections demonstrate their unique value: they preserve physical evidence of past biodiversity, including biological invasions (Holmes et al., 2016; Kim et al., 2023), that may only be recognized and interpreted years later (*cf.* reporting lag, Costello & Solow, 2003), thanks to modern technologies (Boessenkool et al., 2010) and integrative perspectives like ‘collectomics’ (Kapun et al., 2025).

Comparable cases demonstrate how Natural History Collections can reveal overlooked introductions (*e.g.,* Cisneros-Heredia & Peñaherrera Romero, 2020; Northfield, 2024). For instance, museum specimens enabled researchers to revise the invasion chronology of the Harlequin Ladybird *Harmonia axyridis* (Pallas, 1773) in Ecuador, demonstrating that the species arrived earlier than previously documented (Cisneros-Heredia & Peñaherrera Romero, 2020). Yet a key distinction remains: unlike *H. axyridis*’, *A. norrisii*’s case is known from a single individual and shows no evidence of ever having established a self-sustaining population, making any inference about its arrival inherently more uncertain. By contrast, unlike the case of a *Bubopsis* MacLachlan, 1898 larva (Neuroptera, Ascalaphidae) collected in Dorset, UK, in 1984, which benefited from direct clarification by the collector (Northfield, 2024), our interpretation is necessarily constrained by the absence of first-hand testimony.

### An unresolvable matter? Inconclusive particle analysis

**Figure 3:**
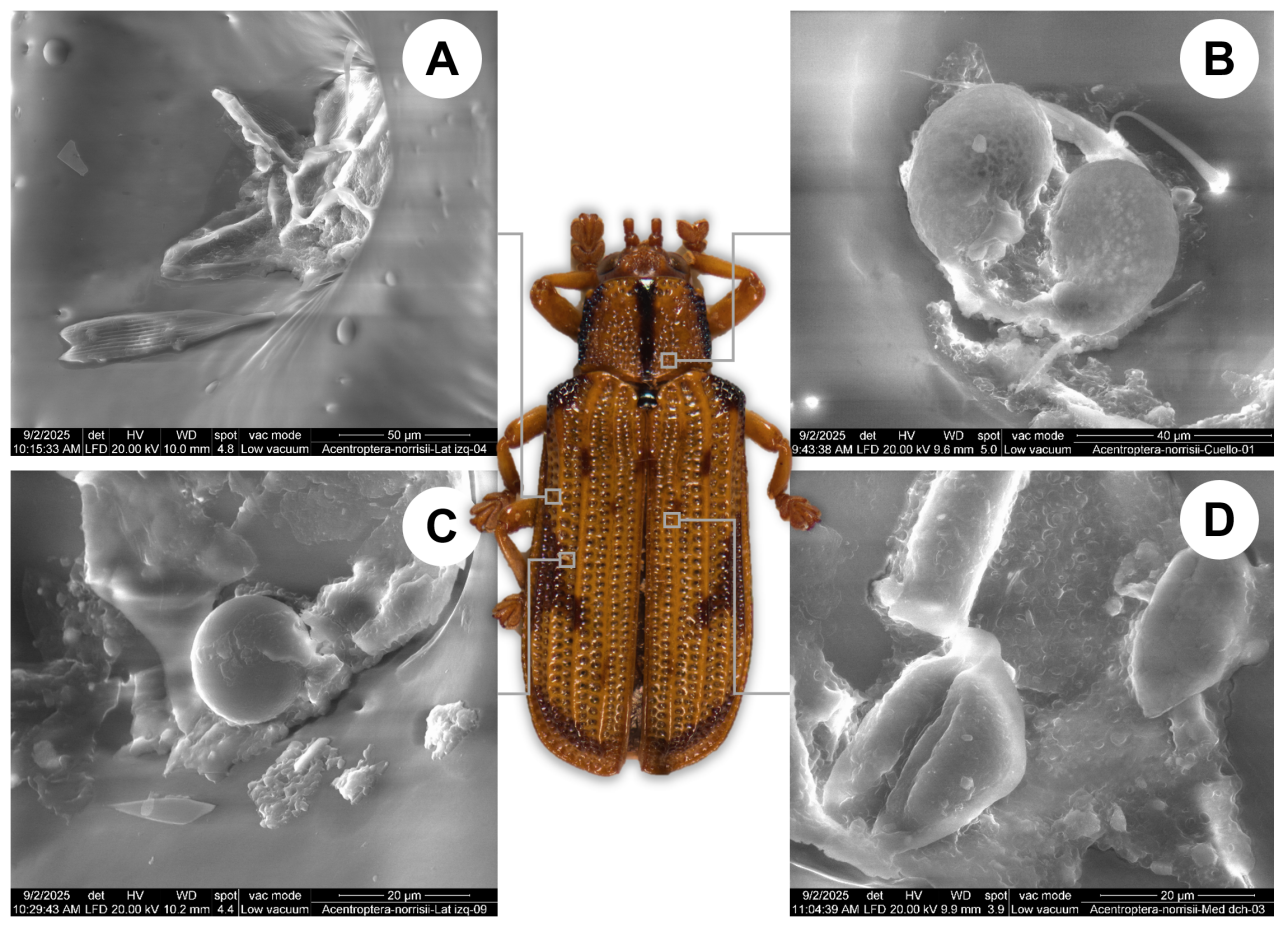
Infographics showing the appearance and location of the particles found in the cuticle of the specimen using SEM. (A) Butterfly wing scale near a collapsed pollen grain. (B) Unique pollen grain corresponding to *Pinus sylvestris* morphotype. (C) One of the pollen grains corresponding to Poaceae morphotype. (D) One of the pollen grains corresponding to Liliaceae morphotype. Photographs by Marta Furió Vega, image assembly by Adrián Sánchez Albert.

Based on the information presented above, the unintentional introduction of *A. norrisii* through international plant trade remains a theoretically plausible scenario. Furthermore, the archival evidence we consulted supports the authenticity of the collection event. However, in order to move beyond speculative and testimonial evidence, we examined the microscopic particles observed on the specimen’s cuticle. The presence of a *Pinus sylvestris* morphotype (Fig. 3B) is particularly relevant, as this species is widespread in the Sierra de Guadarrama (Rivas-Martínez et al., 2002). However, pine pollen is anemophilous, produced in large amounts and capable of travelling long distances (Bohrerova et al., 2009). Consequently, while its presence is compatible with the collection locality, it is also especially susceptible to cross-contamination, reducing its diagnostic value.

The remaining pollen morphotypes (Liliaceae, Poaceae, Figs. 3C and 3D) require caution as well. Many of the specimens stored in the same box were collected within Spanish territory, and several belong to beetle families with well-documented flower-visiting habits (*e.g.,* other Chrysomelidae, Staphylinidae, Cerambycidae, Meloidae; see De Medeiros & Peris, 2025). Although our assessment of the drawer’s content at a relatively coarse taxonomic resolution (family level), it is sufficiently rigorous to raise concern that these pollen morphotypes could also have originated from other anthophilous specimens housed in the same unit. Thus, contamination during storage cannot be fully excluded (Rakosy et al., 2022), which limits the conclusiveness of the palynological evidence.

Nevertheless, certain aspects of the sample support a more cautious, nuanced interpretation. Several pollen grains appear embedded within remains of plant tissue adhered to the cuticle—an arrangement more consistent with direct physical contact on a live plant than with passive deposition during storage (A Sánchez Albert, pers. obs., 2025). Moreover, Rakosy et al. (2022) emphasise that pollen exchange between specimens during storage is generally unlikely, though not impossible. Additional support comes from the presence of an unidentified butterfly wing scale on the beetle (Fig. 3A): to our knowledge, this specimen did not share storage space with Lepidoptera, suggesting that at least part of the adhered material likely originated before curation.

Finally, the diatom (*cf. Planothidium*) found on the cuticle adds little interpretative power, mainly because its ubiquity in soils and freshwater films (Finlay, Monaghan & Maberly, 2002).

## Conclusions

In light of the evidence gathered, we consider it plausible that this single *Acentroptera norrisii* specimen represents a failed historical introduction into Spanish territory rather than a simple labelling error. However, the inconclusive outcome of the particle analysis prevents us of affirming this scenario with total confidence.

This case underscores both the value and the inherent limits of museum-based evidence in invasion biology. With a single, context-poor specimen, a curatorial artefact can hardly be fully ruled out. Yet such uncertainty does not diminish its relevance: if historically uneven occurrences were dismissed outright, much of our understanding of species distributions and introduction histories would be lost. Natural History Collections remain essential precisely because they preserve exceptional material.

Without independent corroboration –such as collector confirmation, further multimedia material (*e.g.,* field photographs) or molecular authentication–, we cannot confidently accept the possibility that this specimen represents a failed biological invasion. Continued work, incorporating molecular approaches will be essential to reduce the uncertainty surrounding this case and fully confirm its accuracy.

## Supplemental Information

10.7717/peerj.21213/supp-1 Supplemental Information 1 Inventory of the specimens that shared the original box with the *Acentroptera norrisii* specimen Specimen counts are organised by taxonomic identity (insect order and family, if known) and by geographical origin, either from Spain or not, based on their label information (if available).

## Additional Information and Declarations

### Data Availability

The following information was supplied regarding data availability: All of the data that support the findings of this study are available in the Materials & Methods and in Fig. 1. For all other specimens sharing the original drawer, please refer to the Supplementary Table. Acentroptera norrisii specimen: MNCN_Ent 438392. The remaining specimens in the original box that were not examined for this work are inventoried and do not yet have their own individual or permanent numbers J. Modolell’s collection specimens are available at the MNCN-CSIC Entomology Collection: ACCESSIONS. Access to the collection can be requested at: https://www.mncn.csic.es/es/colecciones/contacto?col-entomologia@mncn.csic.es.
