# Supplementary material for: The enigmatic historical record of the leaf beetle Acentroptera norrisii in Spain: failed biological introduction or curatorial artefact?
Source: PeerJ. 2026 Jun 18;14:e21213. doi: 10.7717/peerj.21213 (PMC13283361; doi:10.7717/peerj.21213)
Supplement: Supplemental Information 1 — Specimen counts are organised by taxonomic identity (insect order and family, if known) and by geographical origin, either from Spain or not, based on their label information (if available). [file peerj-14-21213-s001.docx]

| **Taxonomic identity** | | **Origin** | | **Specimens per taxa** |
| --- | --- | --- | --- | --- |
| **Order** | **Lower taxa** | **Other** | **Spain** |  |
| **Coleoptera** | **Anthicidae** | 0 | 10 | 10 |
|  | **Bostrichidae** | 3 | 2 | 5 |
|  | **Buprestidae** | 17 | 21 | 38 |
|  | **Cantharidae** | 0 | 5 | 5 |
|  | **Cerambycidae** | 67 | 58 | 125 |
|  | **Chrysomelidae** | 61 | 43 | 104 |
|  | **Cleridae** | 0 | 7 | 7 |
|  | **Coccinellidae** | 9 | 1 | 10 |
|  | **Curculionoidea** | 25 | 30 | 55 |
|  | **Dytiscidae** | 0 | 1 | 1 |
|  | **Elateridae** | 14 | 3 | 17 |
|  | **Erotylidae** | 1 | 0 | 1 |
|  | **Lampyridae** | 2 | 2 | 4 |
|  | **Lycidae** | 10 | 0 | 10 |
|  | **Melandryidae** | 0 | 1 | 1 |
|  | **Meloidae** | 9 | 12 | 21 |
|  | **Melyridae** | 0 | 10 | 10 |
|  | **Ripiphoridae** | 0 | 2 | 2 |
|  | **Staphylinidae** | 8 | 0 | 8 |
|  | **Tenebrionidae** | 16 | 12 | 28 |
|  | **Unknown** | 0 | 3 | 3 |
| **Hemiptera** | | 2 | 1 | 3 |
| **Specimens per origin** | | 274 | 194 | **Absolute total:** 468 |
